# Replacement of miR-155 Elicits Tumor Suppressive Activity and Antagonizes Bortezomib Resistance in Multiple Myeloma

**DOI:** 10.3390/cancers11020236

**Published:** 2019-02-18

**Authors:** Nicola Amodio, Maria Eugenia Gallo Cantafio, Cirino Botta, Valter Agosti, Cinzia Federico, Daniele Caracciolo, Domenica Ronchetti, Marco Rossi, Christoph Driessen, Antonino Neri, Pierosandro Tagliaferri, Pierfrancesco Tassone

**Affiliations:** 1 Department of Experimental and Clinical Medicine, Magna Graecia University of Catanzaro, 88100 Catanzaro, Italy; eugy2186@gmail.com (M.E.G.C.); cirino.botta@gmail.com (C.B.); agosti@unicz.it (V.A.); cinziafederico84@gmail.com (C.F.); mercury86p@gmail.com (D.C.); rossim@unicz.it (M.R.); tagliaferri@unicz.it (P.T.); 2 Department of Oncology and Hemato-oncology, University of Milan and Hematology, Fondazione Cà Granda IRCCS Policlinico, 20122 Milan, Italy; domenica.rochetti@unimi.it (D.R.); antonino.neri@unimi.it (A.N.); 3 Department of Hematology and Oncology, Cantonal Hospital St. Gallen, 9000 St. Gallen, Switzerland; christoph.driessen@kssg.ch; 4 Sbarro Institute for Cancer Research and Molecular Medicine, Center for Biotechnology, College of Science and Technology, Temple University, Philadelphia, PA 19122, USA

**Keywords:** microRNA, miRNA, multiple myeloma, miR-155, bortezomib

## Abstract

Aberrant expression of microRNAs (miRNAs) has been associated to the pathogenesis of multiple myeloma (MM). While miR-155 is considered a therapeutic target in several malignancies, its role in MM is still unclear. The analysis of miR-155 expression indicates its down-regulation in MM patient-derived as compared to healthy plasma cells, thus pointing to a tumor suppressor role in this malignancy. On this finding, we investigated miR-155 replacement as a potential anti-tumor strategy in MM. The miR-155 enforced expression triggered anti-proliferative and pro-apoptotic effects in vitro. Given the lower miR-155 levels in bortezomib-resistant as compared to sensitive MM cells, we analyzed the possible involvement of miR-155 in bortezomib resistance. Importantly, miR-155 replacement enhanced bortezomib anti-tumor activity both in vitro and in vivo in a xenograft model of human MM. In primary MM cells, we observed an inverse correlation between miR-155 and the mRNA encoding the proteasome subunit gene PSMβ5, whose dysregulation has been largely implicated in bortezomib resistance, and we validated PSMβ5 3′UTR mRNA targeting, along with reduced proteasome activity, by miR-155. Collectively, our findings demonstrate that miR-155 elicits anti-MM activity, likely via proteasome inhibition, providing the framework for miR-155-based anti-MM therapeutic strategies.

## 1. Introduction

Multiple myeloma (MM) is a malignancy characterized by accumulation of tumor plasma cells (PCs) in the bone marrow (BM) [1]; here, the interaction between MM cells, extracellular matrix (ECM), and BM stromal cells creates a tumor-promoting milieu, which plays a critical role in supporting cell growth, survival, and drug-resistance [2]. Despite the introduction of new drugs, such as proteasome inhibitors, histone deacetylase inhibitors, and immunomodulatory agents, which have significantly improved the patient clinical outcome, MM still remains an incurable disease [3]. 

MicroRNAs (miRNAs) are small non-coding RNAs of 19-25 nucleotides that regulate gene expression by degradation or translation inhibition of target mRNAs, through complete or partial base pairing in the 3′-untranslated region (3′UTR) [4]. Dysregulated expression of miRNAs has been widely described in solid and hematopoietic malignancies, and has been also recognized as a main player in tumorigenesis [5,6,7,8]. According to their expression levels, aberrantly expressed miRNAs can act as oncogenes (onco-miRNAs), which target and inhibit tumor suppressor genes [9,10,11,12,13,14], or as tumor-suppressors (TS-miRNAs), which negatively regulate oncogenes’ expression [15,16,17,18,19]. Moreover, distinct miRNA signatures have been associated with genetic abnormalities in MM [20], where complex miRNA/transcription factors/target gene regulatory circuits have been identified [21]. Either restoration of TS-miRNAs or inhibition of onco-miRNAs have been proposed as novel experimental approaches, opening new exciting perspectives for the development of miRNA-based therapeutics as single agents, or in combination with current therapeutic regimens in MM [6,7,22,23,24,25,26]. Frequently, miR-155 has been described as being deregulated in human malignancies. It is located on chromosome 21q21.3 and belongs to the B cell integration cluster (BIC) non-coding transcript [27,28]. Despite miR-155 having been found over-expressed in a variety of solid and hematologic malignancies, where it acts as onco-miRNA [29]; very low or null expression was observed in Burkitt lymphoma [30]. In gastric cancer cells, miR-155 inhibits cell metastasis by decreasing SMAD2 expression [31,32]; miR-155 was also found to be expressed in healthy pancreas and absent in endocrine pancreatic tumors [33]. In triple negative breast cancer, upregulation of miR-155 protects against tumor development, and it was included within a specific miRNA signature which correlates with better prognosis [34], inhibits homologous recombination, and enhances cellular sensitivity to ionizing radiation and anti-cancer drugs [35]. The miR-155 deficiency also promoted solid tumor growth by increasing the recruitment and the oncogenic functions of myeloid-derived suppressor cells in the tumor microenvironment [36]. Krzeminski et al. demonstrated methylation of miR-155 locus in MM PCs, accounting for its reduced expression and suggesting a putative oncosuppressive role [37]. Based on these premises we investigated the in vitro and in vivo effects produced by enforced expression of miR-155 on MM cell survival and proliferation; moreover, we explored the ability of miR-155 replacement to overcome bortezomib-resistance. Altogether, our findings unravel a tumor suppressor role of miR-155 in MM, and provide the first framework for potential future applications of miR-155 replacement approaches in the treatment of this malignancy, even in the bortezomib-refractory setting.

## 2. Results

### 2.1. MiR-155 Expression is Dysregulated in MM

**Figure 1:**
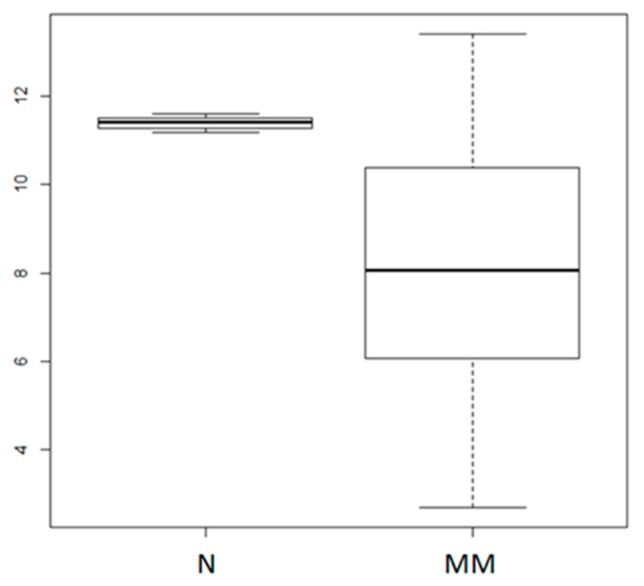
Analysis of miR-155 expression in multiple myeloma primary samples. The miR-155 expression levels in 4 normal donor (N) and 95 primary MM plasma cells (GSE87830); *p* = 0.01684, as calculated by the Wilcoxon rank sum test. Log2 values of normalized miR-155 expression levels are reported on the *y* axis.

We first evaluated miR-155 expression in primary CD138^+^ cells from 4 normal donors and 95 MM patients by analyzing the purified bone marrow CD138^+^ cell fraction of all patients included in a proprietary microarray dataset available through GEO accession number GSE87830 (Figure 1). This analysis indicated miR-155 down-regulation in malignant plasma cells, in agreement with previous work reporting low levels of miR-155 in MM cells [37,38], providing the rationale for studying the effects triggered by miR-155 replacement strategies in MM.

### 2.2. Synthetic miR-155 Mimics Trigger Anti-Proliferative and Pro-Apoptotic Effects in MM Cells

**Figure 2:**
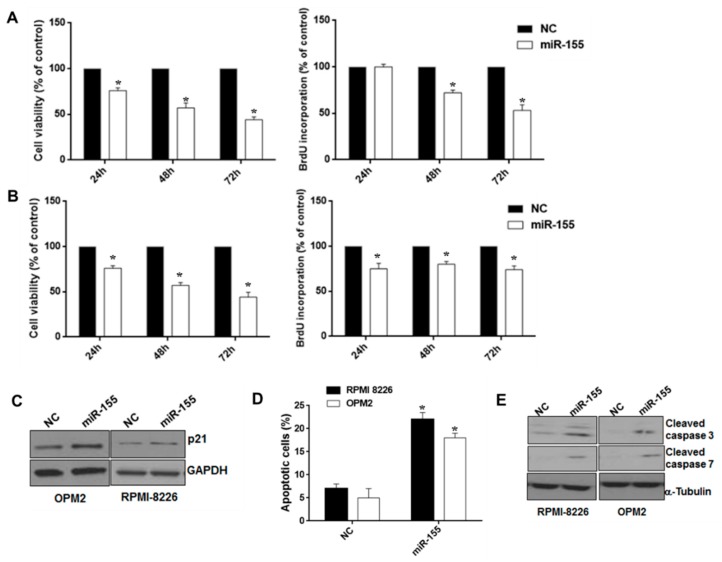
Effects of ectopic miR-155 on MM cell growth, survival, and apoptosis. WST-8 viability and BrdU incorporation assays were performed in RPMI-8226 (**A**) and OPM-2 MM cells (**B**) transfected with synthetic miR-155 (miR-155) or scrambled oligonucleotides (NC) at different time points. Three independent experiments are plotted including ± S.D. (**C**) Immunoblot of p21^CIP1^, 48 hours after transfection of RPMI-8226 and OPM-2 MM cells with synthetic miR-155 or scrambled oligonucleotides (NC). Loading control was performed using GAPDH. (**D**) Annexin V/7-AAD staining performed on RPMI-8226 and OPM-2 cells, 48 hours after transfection with synthetic miR-155 or scrambled oligonucleotides (NC). The percentage of Annexin V-positive cells is reported. Data represent the average of three independent experiments. * *p* < 0.05. (**E**) Immunoblot of cleaved caspase 3 and cleaved caspase 7, 48 hours after transfection of RPMI-8226 and OPM-2 cells with synthetic miR-155 or scrambled oligonucleotides (NC). Loading control was performed using α-tubulin.

To study the function of miR-155, we transfected synthetic miR-155 mimics into MM cell lines expressing low miR-155 levels. Using WST-8 and Bromodeoxyuridine(BrdU) uptake assays, we observed significant inhibition of cell viability and S-phase DNA synthesis induced by miR-155 mimics (Figure 2A,B). Moreover, Western blot (WB) showed that enforced miR-155 enhanced the expression of the cell cycle inhibitor p21^WAF1/CIP1^ in RPMI 8226 and OPM-2 cells, thus suggesting that miR-155 effects on cell growth might be, at least in part, ascribed to cell cycle blockade (Figure 2C); consistently, cell cycle analysis confirmed S phase down-regulation and increase of G0/G1 phase (Figure S1). By Annexin V/7AAD analysis, we further investigated the effects of miR-155 on apoptosis. Indeed, miR-155 mimics increased apoptotic cell death of RPMI-8226 and OPM-2 cells 48 h after transfection, and this event was associated with an increase in caspase 3 and caspase 7 cleaved forms, as demonstrated by WB (Figure 2D,E). Our findings indicate that miR-155 inhibits cells growth and induces apoptosis of MM cells in vitro.

### 2.3. The miR-155 Modulates the Anti-MM Activity of Bortezomib Both In Vitro and In Vivo

**Figure 3:**
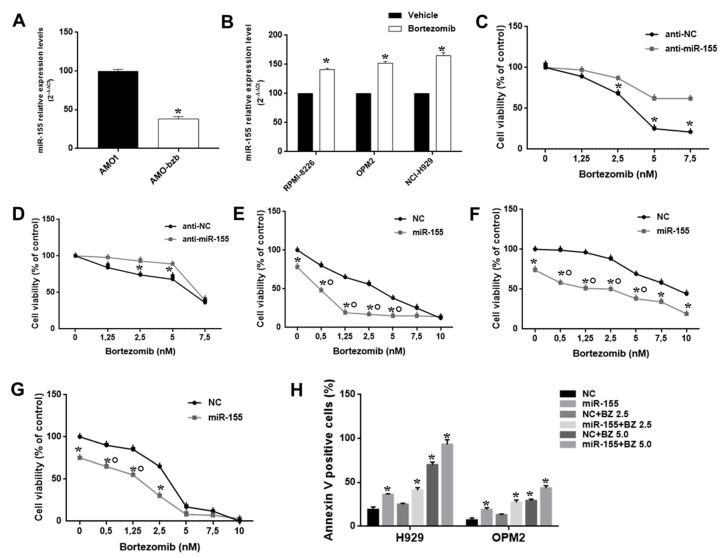
The miR-155 modulates bortezomib anti-MM activity in vitro. (**A**) Quantitative real time-PCR analysis of miR-155 using RNA from AMO-1 and AMO-bzb MM cell lines. Raw Ct values were normalized to RNU44 housekeeping snoRNA and expressed as percentage of miR-155 levels in AMO-1. (**B**) Quantitative RT-PCR analysis of miR-155 using RNA from RPMI-8226, OPM-2, and NCI-H929 cells treated with 2.5 nM bortezomib for 24 h. CCK8 assay performed on NCI-H929 (**C**) and OPM-2 MM cells (**D**) transfected with synthetic miR-155 inhibitors (anti-miR-155) or scrambled oligonucleotides (anti-NC), 48 hours after bortezomib treatment; * *p* < 0.05. CCK8 assay performed on RPMI-8226 (**E**), NCI-H929 (**F**), and OPM-2 MM cells (**G**) transfected with synthetic miR-155 or scrambled oligonucleotides (NC), 48 hours after bortezomib treatment; * *p* < 0.05, indicates synergistic combination (CI>1). (**H**) Apoptosis evaluation of NCI-H929 and OPM-2 MM cells transfected with synthetic miR-155 or scrambled oligonucleotides (NC), 48 h after treatment with different doses of bortezomib; * *p* < 0.05 as compared to NC-transfected cells.

We analyzed miR-155 expression in isogenic AMO1wt and bortezomib-resistant Amo-bzb cell lines. Interestingly, Amo1-bzb displayed lower miR-155 expression as compared to the parental bortezomib-sensitive AMO1 (Figure 3A). Moreover, treatment of MM cells expressing high, intermediate, or low miR-155 levels, namely RPMI-8226, NCI-H929, and OPM-2, with 2.5 nM of bortezomib led to a 30%, 35%, and 40% increase in miR-155 expression, respectively, as shown by qRT-PCR (Figure 3B). Altogether, these findings prompted us to investigate the association between miR-155 expression and sensitivity of MM cells to bortezomib. To address this issue, we first evaluated whether modulation of miR-155 could affect responsiveness of MM cells to bortezomib. NCI-H929 and OPM-2 cells were transfected with miR-155 synthetic inhibitors or scrambled controls, and then cells were exposed to bortezomib. Indeed, we observed that miR-155 inhibition significantly antagonized the growth inhibitory activity of bortezomib (Figure 3C,D).

To further disclose the role of miR-155 in sensitivity of MM cells to bortezomib, its expression was enforced in RPMI-8226, NCI-H929, and OPM2 MM cells by transfection of miR-155 mimics, and 24 h later cells were exposed to increasing bortezomib concentrations. By CCK8 and Annexin V/7-AAD assay, we found that miR-155 mimics potentiated bortezomib activity in terms of both inhibition of cell viability and induction of apoptosis (Figure 3E–H, Figure S2).

**Figure 4:**
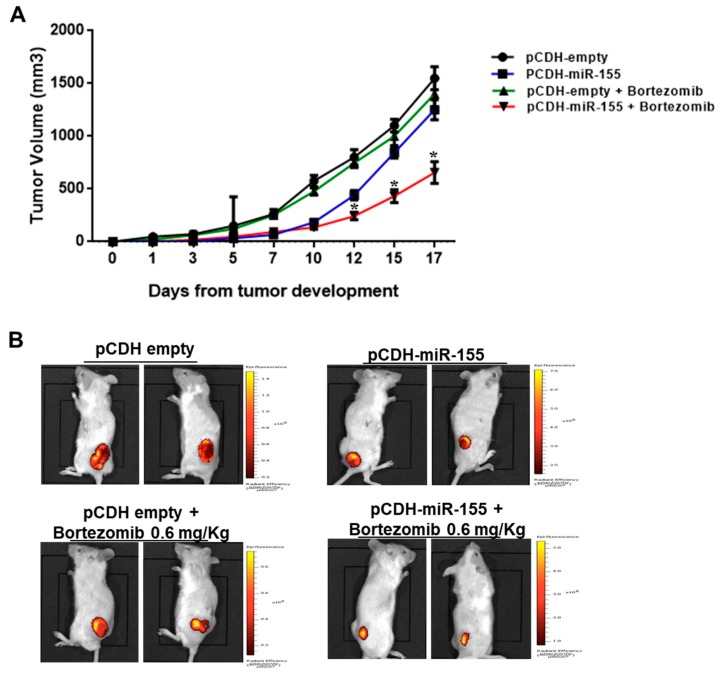
Enforced miR-155 expression enhances bortezomib activity against MM xenografts. (**A**) 2 × 10^6^ of pCDH-miR-155 and 2 × 10^6^ of pCDH-empty vector OPM-2-transduced MM cells were injected, respectively, in the right and left flank of a total of 10 CB-17 NOD-SCID mice. Tumor volume was measured in two dimensions by an electronic caliper, as detailed in materials and methods, every 2–3 days. At day 7, when the tumor volume of each group was approximately 100 mm^3^, mice were treated with 0.6mg/kg of bortezomib; * *p* < 0.05 with respect to pCDH-empty or pCDH plus bortezomib groups. (**B**) Representative IVIS Lumina pictures at day 13 are reported.

We next investigated the effect of miR-155 mimics on the tumorigenic potential of MM cells in vivo. OPM2 cells were transduced with a lentivirus carrying miR-155 (pCDH-miR-155) or with the empty vector (pCDH-empty), and next inoculated subcutaneously in the right and left flank of mice, respectively; tumor formation was monitored over time. When tumor volume was almost 100 mm^3^ for each group (approximately 7 days after tumor formation), mice were intraperitoneally injected with 0.6 mg/Kg of bortezomib or saline. As shown in Figure 4A, stable miR-155 expression enhanced bortezomib-induced growth inhibition of MM xenografts as compared to controls (*p* < 0.05). WB analysis of lysates from explanted MM xenografts confirmed PSMβ5 down-regulation in miR-155-transduced MM xenografts (Figure S3).

### 2.4. miR-155 Targets the Proteasome Subunit β5 (PSMβ5) and Sensitizes AMO-bzb Cell Line to Bortezomib

**Figure 5:**
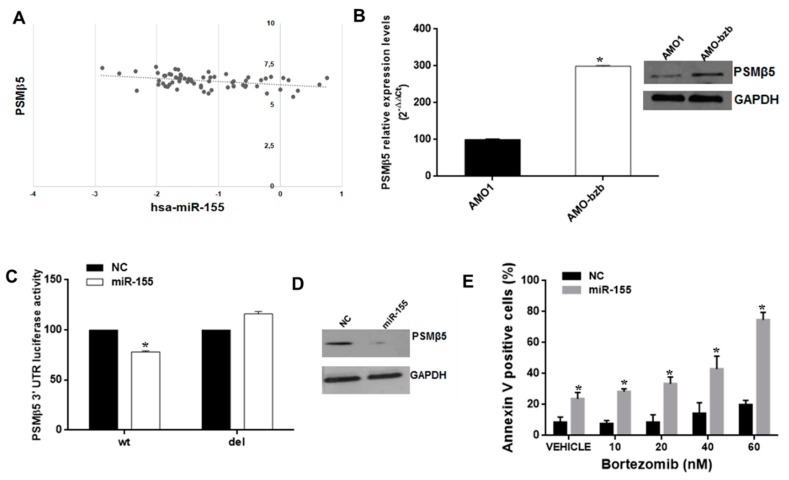
The miR-155 targets PSMβ5 and sensitizes the AMO-bzb cell line to bortezomib. (**A**) Inverse correlation between miR-155 and PSMB5 levels in MM patient plasma cells (Gene Expression Omnibus accession number GSE16558; *R*^2^ = 0.15, *p* = 0.0015). (**B**) Quantitative RT-PCR and WB analysis of PSMβ5 using total RNA or total protein lysates respectively from AMO1 and AMO-bzb. Raw Ct values were normalized to GAPDH housekeeping gene and expressed as percentage of AMO1 cell line. Values represent mean of three different experiments. For the immunoblot of PSMβ5 in AMO1 and AMO-bzb, GAPDH was used as loading control. (**C**) Dual luciferase assay in AMO-bzb cells co-transfected with firefly luciferase constructs containing the 3′UTR of PSMβ5 or a deleted 3′UTR construct, and miR-155 or scrambled oligonucleotides (NC) as indicated. The firefly luciferase activity was normalized to renilla luciferase activity. The data are shown as relative luciferase activity of miR-155-transfected cells as compared to the control (NC) of a total of six experiments from three independent transfections. (**D**) Immunoblot of PSMβ5 in AMO-bzb MM cells, 72 h after transfection with synthetic miR-155 or scrambled oligonucleotides (NC). Loading control was performed using GAPDH. (**E**) Apoptosis evaluation of AMO-bzb cells transfected with synthetic miR-155 or scrambled oligonucleotides (NC), 72 h after treatment with different doses of bortezomib; * *p* < 0.05 as compared to NC-transfected cells.

Molecular mechanisms underlying bortezomib resistance are diverse, and include, among others, mutations or upregulation of PSMβ5 [7]. We searched for proteasome-associated genes potentially targeted by miR-155. Interestingly, interrogation of the microRNA.org database revealed that miR-155 is predicted to target the 3′UTR of PSMβ5 mRNA. Therefore, we explored whether miR-155 and PSMβ5 levels were correlated in MM patients. Notably, we found that miR-155 and PSMβ5 mRNA levels were inversely correlated in a publicly available MM dataset (GEO accession number GSE16558; *p* = 0.001542; Figure 5A); the same inverse correlation could be observed in isogenic AMO1 cells. Indeed, the bortezomib-resistant AMO1-bzb cell line showed increased expression of PSMβ5 mRNA and protein (Figure 5B), while it expressed lower miR-155 levels as compared to the parental counterpart (Figure 3A). By a luciferase reporter assay, we investigated whether miR-155 could directly target the 3′UTR of PSMβ5 mRNA. To this purpose, AMO-bzb cells were co-transfected with a luciferase construct containing the 3′UTR of PSMβ5, or with a 3′UTR deletion mutant lacking the predicted miR-155 target site, together with miR-155 mimics or scrambled oligonucleotides (NC). We observed reduced luciferase activity in cells treated with miR-155 mimics as compared to control; no significant change was observed when the mutant 3′UTR luciferase construct devoid of the miR-155 target sequence was used. We also observed reduced PSMβ5 protein abundance in cells transfected with miR-155 mimics (Figure 5D). To assess whether miR-155 mimics could restore bortezomib sensitivity, AMO-bzb cells were transfected with synthetic miR-155 or negative control (miR-NC), and then treated with increasing bortezomib concentrations. Annexin V/7AAD staining performed after 72h indicates restored sensitivity to bortezomib in cells treated with synthetic miR-155 mimics, as evidenced by significant increase in the percentage of apoptotic cells upon miR-155 mimics plus bortezomib treatments (Figure 5E and Figure S4).

### 2.5. PSMβ5-Silencing Phenocopies miR-155 Effects on Bortezomib Sensitivity

**Figure 6:**
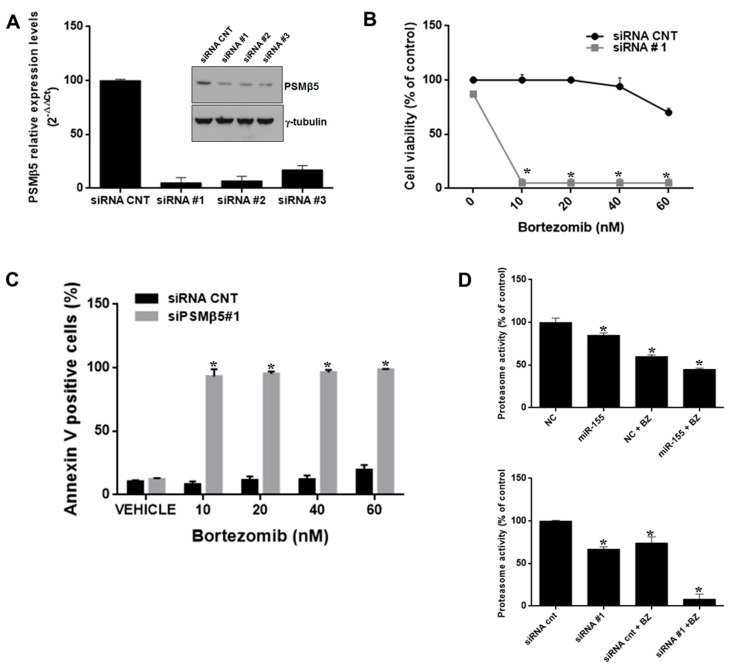
PSMβ5 inhibition sensitizes AMO-bzb cells to bortezomib. (**A**) Quantitative RT–PCR and WB analysis of PSMβ5 in AMO-bzb MM cells transfected with synthetic siRNA control (siRNA CNT) or three different siRNAs (siRNA#1, siRNA#2, siRNA#3) targeting PSMβ5. Raw Ct values were normalized to GAPDH and expressed as percentage of AMO-bzb cell line transfected with NC. Values represent mean of three different experiment. CCK8 assay (**B**) and Annexin V staining (**C**) of AMO-bzb MM cells transfected with synthetic siRNA control (siRNA CNT) or siRNA#1, 72 h after bortezomib treatment at final concentration of 10 nM, 20 nM, 40 nM, and 60 nM respectively; * *p* < 0.05. (**D**) Proteasome activity assay was performed on AMO-bzb MM cells transfected with synthetic miR-155 or scrambled oligonucleotides (NC), synthetic siRNA control (siRNA CNT), or siRNA#1, and then treated for 24 h with 2.5 nM bortezomib. NC vs miR-155, * *p* < 0.05; NC vs NC+2.5 nM Bortezomib, * *p* < 0.05; NC+2.5 nM Bortezomib vs miR-155 + 2.5 nM Bortezomib, * *p* < 0.05; siRNA CNT vs siRNA #1, * *p* < 0.05; siRNA CNT vs siRNA CNT+2.5 nM bortezomib, * *p* < 0.05; siRNA CNT+2.5 nM bortezomib vs siRNA#1 +2.5 nM bortezomib, * *p* < 0.05.

Finally, we tested whether PSMβ5-silencing could phenocopy miR-155-induced effects on MM cells. To this aim, we first transfected AMO-bzb cells with synthetic siRNA control or with 3 different synthetic PSMβ5-targeting siRNAs. As shown in Figure 6A, PSMβ5 was downregulated both at the mRNA and protein level (Figure 6A). We observed strong inhibition of cell viability (Figure 6B) and apoptosis induction (Figure 6C, Figure S5 in the Supplementary Materials) in AMO-bzb cells transfected with synthetic siRNA#1 and treated with bortezomib, confirming that PSMβ5 down-regulation sensitizes bortezomib-resistant MM cells to the drug. Conversely, PSMβ5 silencing did not induce any change in miR-155 expression (data not shown), thus ruling out the occurrence of a regulatory feedback loop between the two molecules. Next, we investigated the effects of bortezomib/miR-155 or bortezomib/PSMβ5 siRNA combinations on β5 proteasome activity. AMO-bzb cells were electroporated with synthetic miR-NC or miR-155, or synthetic siRNA CNT or PSMβ5 siRNA, and then treated with 2.5 nM of bortezomib; proteasome activity was measured 48 h after transfection. As shown in Figure 6D, miR-155 was enhanced by 20% in bortezomib-induced proteasome inhibition, while the PSMβ5-targeting siRNA#1 was enhanced by 66%. All together, these findings indicate that miR-155 restores the drug responsiveness of bortezomib-resistant MM cells in vitro, likely via proteasome inhibition, and that PSMβ5 silencing recapitulates the same phenotypic effects.

## 3. Discussion

In this report, we demonstrate that miR-155 is dysregulated in MM, acts as a tumor suppressor, and plays a mechanistic role in MM cell responsiveness to bortezomib.

It is known that miR-155 is a well-studied miRNA, acting as an oncomiRNA in many solid and hematologic tumors, where it was found to be overexpressed, enhanced proliferation, and antagonized chemotherapy [39,40]. However, it has recently emerged that high levels of miR-155 correlate with longer overall survival, and its expression decreases in relapsed disease as compared to diagnosis in MM patients [20]. Importantly, hypermethylation of the first exon of miR-155 host gene accounted for its reduced expression in MM cells [37], as further confirmed in a genome-wide screening for potential tumor suppressive miRNAs epigenetically silenced in MM [38].

In the present study, we extended previous findings on reduced expression of miR-155 in MM patient-derived CD138^+^ PCs [37,38]. Very recent multi parameter flow cytometry-based analyses of PC disorders indicate that these cell fractions might be likely contaminated by normal CD138^+^ PCs [41]; miR-155 appeared to be significantly lower in MM PCs than in normal controls. Importantly, our data highlighted for the first time the therapeutic potential of miR-155 restoration by using established in vitro and in vivo preclinical models of this malignancy. In fact, miR-155 mimics transfection hampered cell cycle progression and triggered apoptotic cell death in vitro. Moreover, the observation that miR-155 expression levels are reduced in bortezomib-resistant cells as compared to the wild-type counterpart, prompted us to investigate the impact of miR-155 modulation on bortezomib anti-tumor activity.

Bortezomib (PS-341) is the prototype of proteasome inhibitors with potent anti-MM activity. It is commonly used to treat newly diagnosed or relapsed MM patients, but the development of off-target toxicities and drug-resistance limits its use in the long-term [42,43]. So far, the investigation of molecular mechanisms underlying bortezomib resistance [44] has taken advantage of resistant cell lines generated by stepwise exposure to increasing concentrations of bortezomib. Altogether, these studies have provided evidence that dysregulation of miRNAs-[19,45,46], long non-coding RNAs-[47,48], or protein-coding gene-regulated pathways can likely contribute to the onset of bortezomib resistance. 

In MM cells, we previously demonstrated that bortezomib treatment induces a “BRCAness” status which leads to the repression of genes involved in homologous recombination, including BRCA1 [49]. Since BRCA1 was found to epigenetically repress miR-155 [50], we wondered whether bortezomib could promote miR-155 expression via BRCA1 inhibition. To address this hypothesis, we preliminarily analyzed if BRCA1 mRNA levels correlated with those of miR-155 in our microarray dataset (GSE87830), although no significant association could emerge (data not shown), suggesting that additional, as yet undetermined mechanisms might be involved in bortezomib-induced miR-155 upregulation. Future studies will be performed to shed light on this point. 

We next attempted to identify molecular targets explaining miR-155 role in MM cells; to this aim, we interrogated the microRNA.org database, and identified the proteasome subunit PSMβ5 as a predicted miR-155 target.

Single point mutations in PSMβ5, causing conformational or steric changes to the proteasome drug-binding site, have been described as a major mechanism underlying bortezomib resistance [51], along with downregulation of 19S proteasome subunits [52,53], overexpression of PSMβ5 without the occurrence of mutations [51], increase in drug transporters [54], or in microenvironmental proteins and chaperones [55].

We indeed validated for the first time the 3′UTR targeting of PSMβ5 mRNA by miR-155, and we found an inverse correlation between miR-155 and PSMβ5 on a public microarray dataset of primary MM cells, for which both miRNA and gene expression profiling data were available. Collectively, these findings led us to speculate that miR-155 could play a role in bortezomib-resistance. In line with this hypothesis, we found that miR-155 overexpression decreased PSMβ5 protein levels, thus leading to reduced proteasome activity and increased sensitivity of MM cells to the drug; on the other hand, miR-155 inhibitors antagonized the effects of bortezomib on drug-sensitive cells in vitro. Of note, silencing of PSMβ5, which was overexpressed in AMO-bzb-resistant cells as compared to the parental cell line, phenocopied miR-155 replacement and potently sensitized, even at higher extent than miR-155 overexpression, drug resistant cells to anti-tumor activity of bortezomib. Our findings therefore strongly support the concept that PSMβ5 dysregulation plays a critical role in bortezomib-resistance mechanisms, and provide at our knowledge the first evidence that miR-155, by targeting PSMβ5, may antagonize bortezomib resistance of MM cells; however, taking into account the intrinsic pleiotropic function of miRNAs, we cannot rule out that further oncogenic pathways could be antagonized by miR-155 to elicit its tumor suppressive activity.

Finally, we provided novel evidence of bortezomib/miR-155 positive interaction in vivo, as demonstrated by superior anti-tumor activity of the proteasome inhibitor against human MM xenografts stably expressing miR-155 as compared with controls. Altogether, these data underscore the effectiveness of miR-155 reconstitution to enhance or restore bortezomib sensitivity of MM cells, offering the rationale for a novel translational path.

## 4. Materials and Methods

### 4.1. Cell Lines and Drugs

Human MM cell lines NCI-H929, RPMI-8226, U266, OPM-2, MM1s, and AMO-1 were purchased from DSMZ (Braunschweig, Germany), which certified authentication performed by short tandem repeat DNA typing. KMS11 cell line was obtained by Japanese Collection of Research Bioresources (National Institute of Health Sciences Japan, Osaka, Japan). AMO-1 wild type and bortezomib resistant AMO-bzb cells were previously described [56]. All these cell lines were immediately frozen and used from the original stock within 6 months. MM cell lines were cultured in RPMI- 1640 medium (Gibco®, Life Technologies, Carlsbad, CA, USA), supplemented with 10% fetal bovine serum (Lonza GroupLtd., Basel, Switzerland), 100 U/mL penicillin, and 100 mg/mL streptomycin (Gibco®, Life Technologies), and maintained at 37 °C in a 5% CO_2_ atmosphere. Cells stably expressing pre-miR-155 were generated using pCDH-CMV-MCS-EF1-GFP-Puro lentivector plasmid or the corresponding empty vector (purchased from SBI System Biosciences, Mountain View, CA, USA). Lentivirus was produced in HEK 293T packaging cells, as described [44]; two rounds of transduction of MM cells in the presence of 8 μg/mL of polybrene (Sigma Sigma-Aldrich, St. Louis, MO, USA) were performed. Two days after transduction, cells were selected in medium containing 1 μg/ml Puromycin (Sigma-Aldrich). Clinical grade bortezomib for in vivo experiments was obtained from Millennium Pharmaceuticals (Takeda, Cambridge, MA, USA) and dissolved in sodium chloride saline solution at 1 mg/mL; for in vitro studies, bortezomib was purchased from Selleck chemicals (Houston, TX, USA) and dissolved in DMSO. 

### 4.2. Quantitative Real-Time Amplification of miRNAs and mRNAs

Total RNA from MM cells and peripheral blood mononuclear cells (PBMCs) was prepared with the TRIzol® Reagent (Invitrogen, Thermo Scientific, Carlsbad, CA, USA) according to manufacturer’s instructions. Oligo-dT-primed cDNA was obtained using the High Capacity cDNA Reverse Transcription Kit (Applied Biosystems, Thermo Scientific, Carlsbad, CA, USA). The single-tube TaqMan miRNA assays were used to detect and quantify mature miR-155 and target mRNAs according to the manufacturer’s instructions by the use of the StepOne Thermocycler and the sequence detection system (Applied Biosystems). The miR-155 and mRNAs were normalized on RNU44 and GAPDH (Ambion, Carlsbad, CA, USA), respectively. Comparative real-time polymerase chain reaction (RT-PCR) was performed in triplicate. Relative expression was calculated using the comparative cross threshold (ΔΔCt) method as described [57].

### 4.3. In Vitro Transfection of MM cells

1 × 10^6^ MM cells were electroporated with scrambled (miR-NC), synthetic pre-miR-155 (miR-155), siRNA control (siRNA CNT), and three different siRNAs against PSMβ5 (Invitrogen, Thermo Scientific, Invitrogen, Thermo Scientific, Carlsbad, CA, USA), at a final concentration of 100 nM, using Neon® Transfection System (Life Technologies) at 1150 V, 30 ms, 2 pulse setting.

### 4.4. CCK8, BrdU, and Cell Cycle Assays

Cell viability was evaluated by WST-8 assay using the Cell Counting Kit-8 (CCK-8 Dojindo), and by BrdU proliferation assay (DELFIA cell proliferation assay, PerkinElmer, DELFIA cell proliferation assay, PerkinElmer, Waltham, MA, USA. Electroporated cells were incubated for 1 h in 6-well plates; after harvesting, cells were seeded in 96-well plates for CCK-8 and BrdU proliferation assay. CCK8 was measured every 24 h and the optical density (OD) was evaluated at the 450 nm wavelength. BrdU uptake was also measured every 24 h, and luminescence detected using a Victor4 plate reader (PerkinElmer). Each sample was run at least in quadruplicate. To assess synergism between miR-155 and bortezomib, Combination Index (CI) was calculated by the following formula: CI = (inhibition COMBO)/(inhibition miR-155 mimics) + (inhibition bortezomib), where CI > 1, CI = 1, and CI < 1 indicate synergistic, additive, and antagonistic effects, respectively.

To evaluate alterations in cell cycle, FACS analysis was performed on transfected MM cells after staining with Propidium Iodide (PI). In detail, cells were collected, washed twice with phosphate-buffered saline (PBS) and fixed in cold 70% ethanol at 20 °C. Before FACS analysis, cells were washed with PBS and stained in 50 μg/mL PI, 100 μg/mL RNase, 0.05% Nonidet P-40 for 1 h at room temperature in the dark. Cell cycle profiles were determined using FCS Express 6 software (De Novo Software, Los Angeles, CA, USA). 

### 4.5. Luciferase Reporter Experiments

The wild-type 3′UTR of PSMβ5 gene containing the predicted target site for miR-155 and the deletion mutant construct lacking the site of interaction (nucleotides from 130 to 180) were cloned in pEZX-MT01 vector and purchased from Genecopoeia. MM cells were electroporated, as described above, using 5 μg of the luciferase reporter vector; for each plate, 100 nM of the synthetic miR-155 or scrambled (miR-NC) were used. Firefly and renilla luciferase activities were measured consecutively using the dual-luciferase assay kit (Promega Corporation, Madison, WI, USA), 24 hours after transfection in RPMI-8226 cells, using GloMAX (Promega). Data are expressed as the ratio between the firefly luminescence and the renilla luminescence.

### 4.6. Western Blotting (WB) Analysis

SDS-PAGE and WB were performed according to standard protocols, as described [58]. Briefly, cells were lysed in lysis buffer containing 15 mM Tris/HCl pH 7.5, 120 mM NaCl, 25 mM KCl, 1 mM EDTA, 0.5% Triton 100, Halt Protease Inhibitor Single-Use cocktail (100×, Thermo Scientific). Whole cells lysates (50 μg per sample) from transfected cell lines were separated using 4–12% Novex Bis-Tris SDS-acrylamide gels (Invitrogen), electro-transferred on nitrocellulose membranes (Bio-Rad, Hercules, CA, USA), and immunoblotted with the following antibodies: PSMβ5 (D1H6B) Rabbit mAb (Cell Signaling), Caspase-3 (8G10) Rabbit mAb (Cell Signaling), Caspase-7 (C7) Mouse mAb (Human Specific), γ-Tubulin antibody (C-20) goat polyclonal (Santa Cruz Biotechnology, Santa Cruz, CA, USA), and GAPDH (D16H11) XP® Rabbit mAb. Membranes were washed 3 times in PBS-Tween, and then incubated with a secondary antibody conjugated with horseradish peroxidase in 5% milk for 2 h at room temperature. Chemiluminescence was detected using Pierce ECL Western Blotting Substrate (Pierce Biotechnology, Waltham, MA, USA). 

### 4.7. Animals and In Vivo Model of Human MM 

Male CB-17 severe combined immunodeficient (SCID) mice (6- to 8-weeks old; Harlan Laboratories, Inc., Indianapolis, IN, USA) were housed and monitored in our Animal Research Facility. All experimental procedures had been conducted according to protocols approved by the Italian Ministry of Health (authorization n. 126/2016-PR). In accordance with institutional guidelines, mice were sacrificed when their tumors reached 2 cm in diameter or in the event of paralysis or major compromise in their quality of life, to prevent unnecessary suffering. For these studies, SCID mice were inoculated subcutaneously on the right and left flank with 2 × 10^6^ of transduced MM cells (in 100 μL culture medium) stably expressing miR-155 or empty vector by lentivirus-based transduction; 5 mice/group were used. Tumor sizes were measured every two days in two dimensions using a caliper, and volume was calculated using the formula V = 0.5 × a × b^2^, where a and b are the long and short diameter of the tumor, respectively. The IVIS Lumina ΙΙ (Caliper LS, Hopkinton, MA, USA) instrument was used for generating optical images by Living Images software equipped with the GFP (green fluorescent protein) filter.

### 4.8. Proteasome Activity Assay

Proteasome activity assay kit (ab107921) was purchased from abcam (Cambridge, UK); 1 × 10^6^ AMO-bzb MM cells were electroporated with 100 nM of miR-NC or miR-155, siRNA CNT, or siRNA targeting PSMβ5; bortezomib was added 24 h after electroporation, at the final concentration of 2.5 nM. Proteasome activity was determined 48 h after electroporation, according to the manufacturer’s instructions.

### 4.9. Statistical Analysis

Each experiment was performed at least three times, and values were reported as mean ± SD. Data were analyzed using Student’s *t* tests for two group comparisons or a one-way analysis of variance (ANOVA) for multiple comparisons using the Graphpad software (GraphPad Software, La Jolla, CA, USA); *p*-value  <  0.05 was considered significant.

## 5. Conclusions

Our data demonstrate that in MM miR-155, in contrast with other types of cancers where it acts as onco-miRNA, exerts a protective role against tumorigenesis and alleviates bortezomib resistance, likely via PSMβ5 targeting. Taken together, these findings pave the way for potential miR-155-based therapeutic strategies against this still fatal disease, especially in the relapsed and refractory setting.

## Supplementary Materials

The following are available online at http://www.mdpi.com/2072-6694/11/2/236/s1, Figure S1: miR-155 overexpression in MM cells impairs the cell cycle. RPMI-8226 cells were transfected with 100 nM of miR-155 mimics or scrambled control oligonucleotides (NC), and cell cycle was then evaluated by PI staining and FACS analysis as reported in Materials and Methods. * *p* < 0.05. Figure S2: miR-155 overexpression triggers apoptosis of MM cells. OPM2 and NCI-H929 were transfected with 100 nM of syntethic miR-155 or scrambled oligonucleotides (NC), and then treated with different doses of bortezomib for 48 hours, as indicated in each plot. Annexin V/7AAD staining and FACS analysis were then performed. A representative experiment is reported. Figure S3: miR-155 overexpression reduces PSMβ5 expression in MM xenografts. WB analysis of PSMβ5 protein in lysates from OPM2 xenografts expressing pCDH-empty vector or pCDH-miR-155, explanted at day 17. GAPDH was used as loading control. Figure S4: miR-155 overexpression alleviates bortezomib resistance in MM cells. AMO-bzb cells were transfected with 100 nM syntethic miR-155 or scrambled oligonucleotides (NC), and then treated with different doses of bortezomib for 72 hours, as indicated in each plot. Annexin V/7AAD staining and FACS analysis were then performed. A representative experiment is reported. Figure S5: PSMβ5 silencing alleviates bortezomib resistance in MM cells. AMO-bzb cells were transfected with 100 nM syntethic PSMβ5 siRNAs (siRNA #1) or siRNA control (siRNA CNT), and then treated with different doses of bortezomib for 72 hours, as indicated in each plot. Annexin V/7AAD staining and FACS analysis were then performed. A representative experiment is reported.
