# Supplementary material for: Replacement of miR-155 Elicits Tumor Suppressive Activity and Antagonizes Bortezomib Resistance in Multiple Myeloma
Source: Cancers (Basel). 2019 Feb 18;11(2):236. doi: 10.3390/cancers11020236 (PMC6406286; doi:10.3390/cancers11020236)
Supplement: Supplementary file 1 [file cancers-11-00236-s001.pdf]

# Supplementary Materials: Replacement of miR-155 Elicits Tumor Suppressive Activity and Antagonizes Bortezomib Resistance in Multiple Myeloma

**Nicola Amodio, Maria Eugenia Gallo Cantafio, Cirino Botta, Valter Agosti, Cinzia Federico, Daniele Caracciolo, Domenica Ronchetti, Marco Rossi, Christoph Driessen, Antonino Neri, Pierosandro Tagliaferri and Pierfrancesco Tassone**

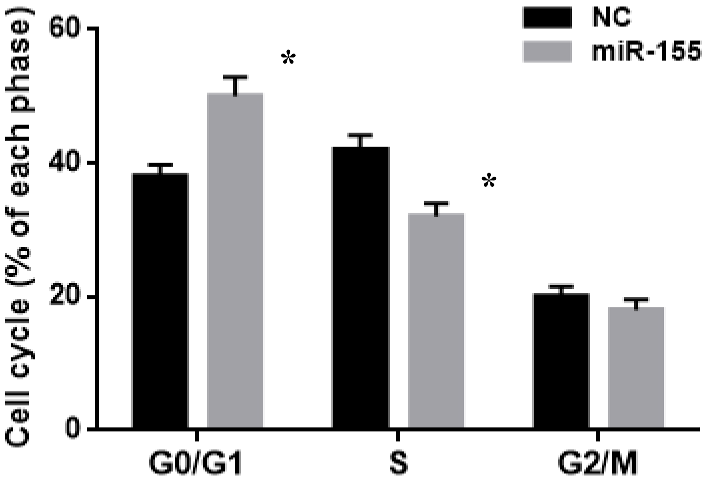


**Figure S1.** MiR-155 overexpression in MM cells impairs the cell cycle. RPMI-8226 cells were transfected with 100nM of miR-155 mimics or scrambled control oligonucleotides (NC), and cell cycle was then evaluated by PI staining and FACS analysis as reported in Materials and Methods. *$p < 0.05$.

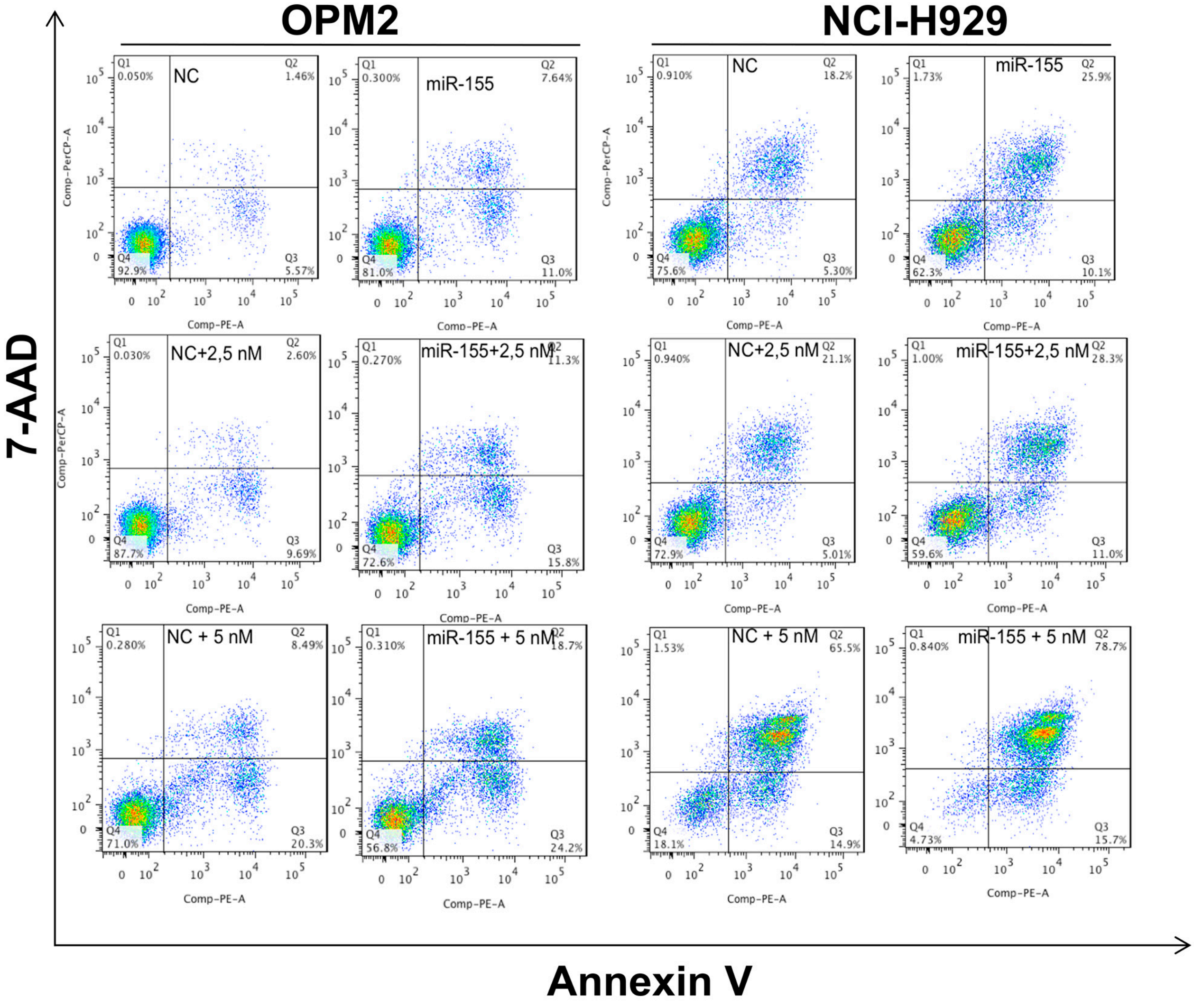


**Figure 2.** MiR-155 overexpression triggers apoptosis of MM cells. OPM2 and NCI-H929 were transfected with 100nM of syntethic miR-155 or scrambled oligonucleotides (NC), and then treated with different doses of bortezomib for 48 hours, as indicated in each plot. Annexin V/7AAD staining and FACS analysis were then performed. A representative experiment is reported.

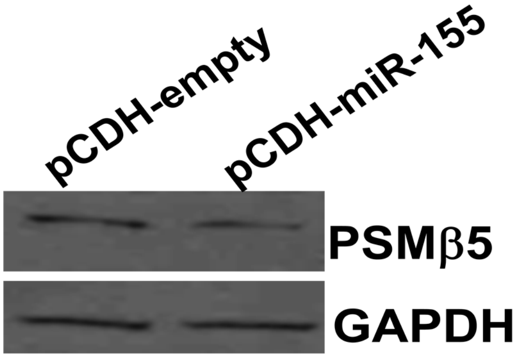


**Figure 3.** MiR-155 overexpression reduces PSMβ5 expression in MM xenografts. WB analysis of PSMβ5 protein in lysates from OPM2 xenografts expressing pCDH-empty vector or pCDH-miR-155, explanted at day 17. GAPDH was used as loading control.

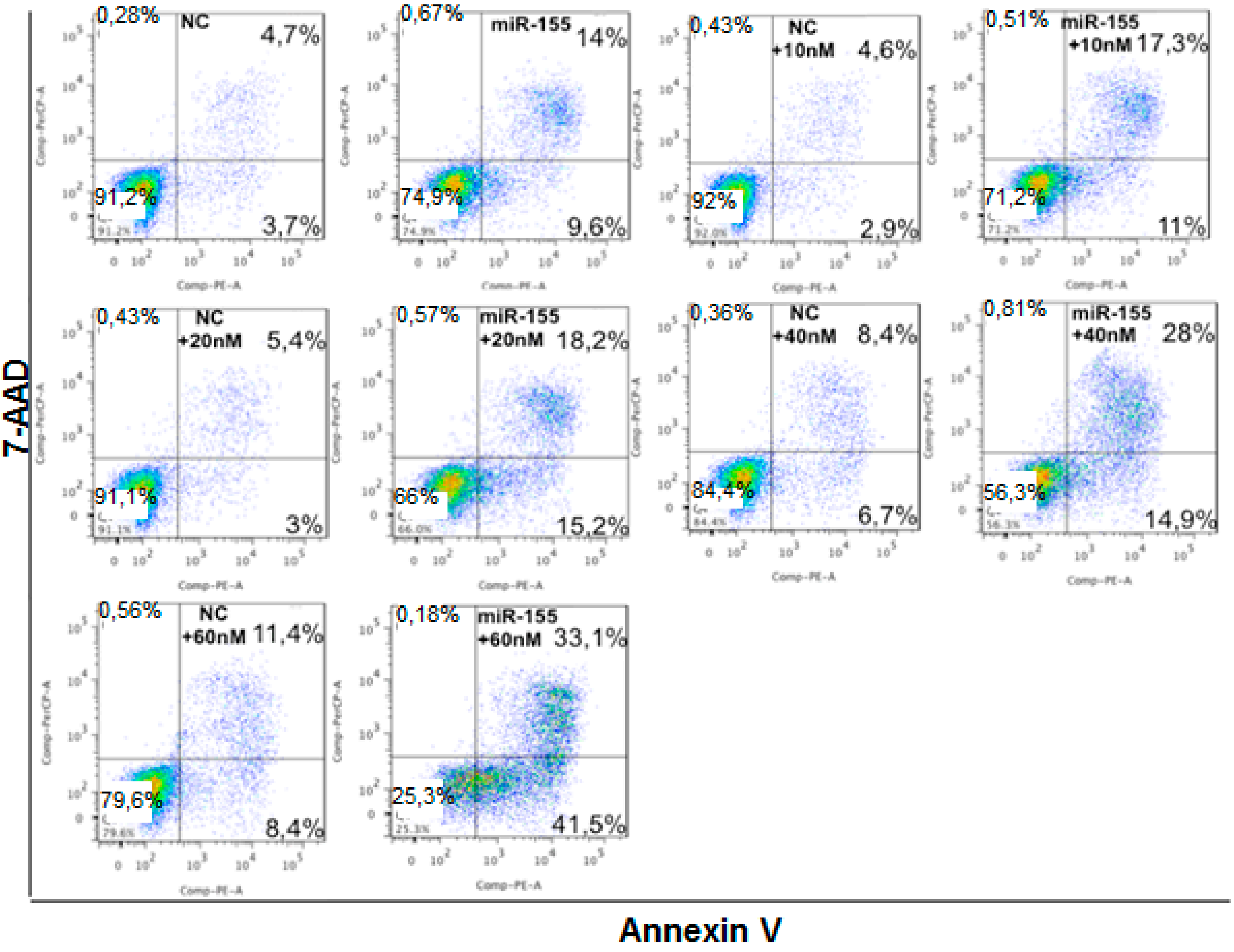


**Figure 4.** MiR-155 overexpression alleviates bortezomib resistance in MM cells. AMO-bzb cells were transfected with 100nM synthetic miR-155 or scrambled oligonucleotides (NC), and then treated with different doses of bortezomib for 72 hours, as indicated in each plot. Annexin V/7AAD staining and FACS analysis were then performed. A representative experiment is reported.

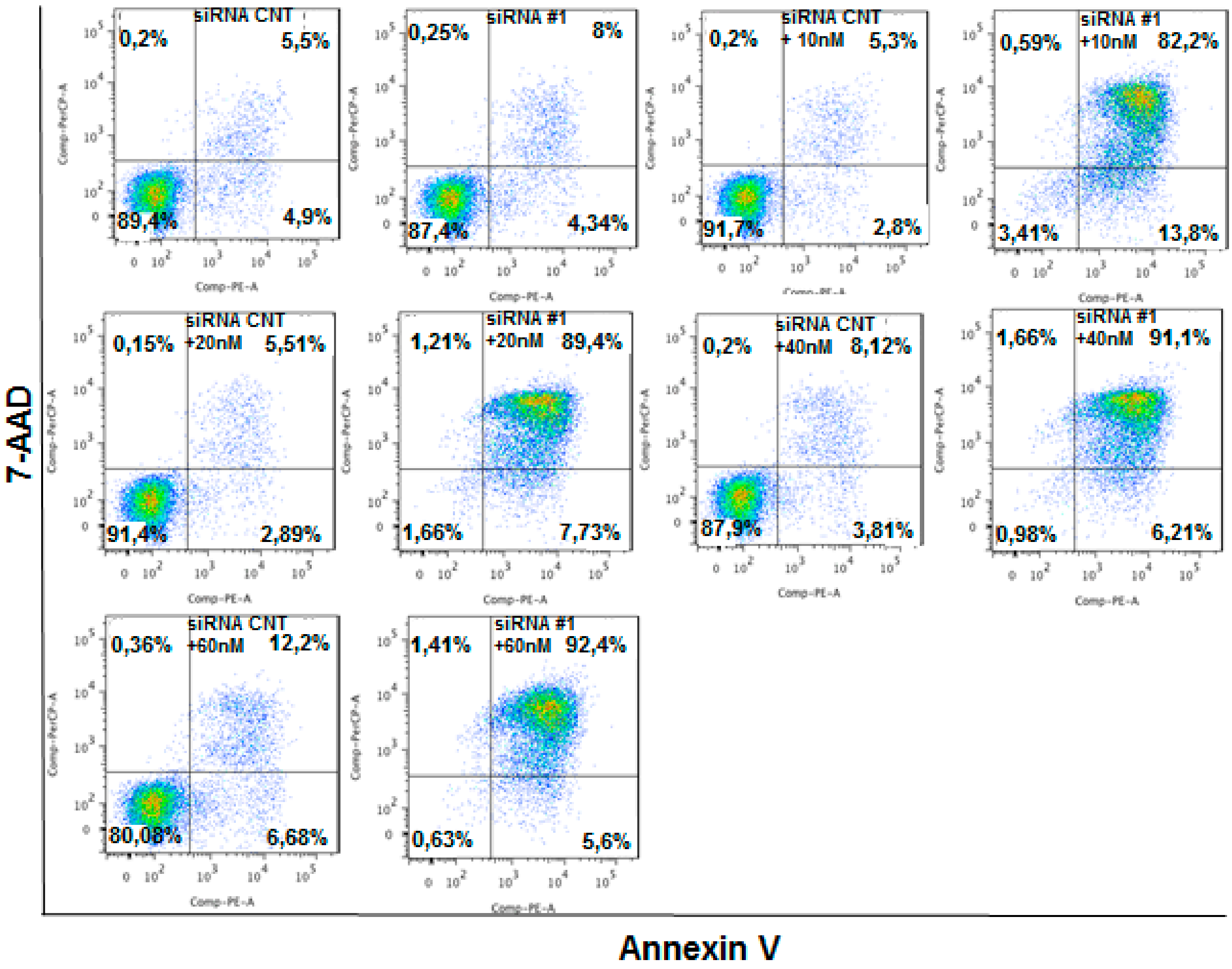


**Figure 5.** PSMβ5 silencing alleviates bortezomib resistance in MM cells. AMO-bzb cells were transfected with 100nM synthetic PSMβ5 siRNAs (siRNA #1) or siRNA control (siRNA CNT), and then treated with different doses of bortezomib for 72 hours, as indicated in each plot. Annexin V/7AAD staining and FACS analysis were then performed. A representative experiment is reported.
